# Epigenetic control of gene function in schistosomes: a source of therapeutic targets?

**DOI:** 10.3389/fgene.2014.00317

**Published:** 2014-09-10

**Authors:** Alejandro Cabezas-Cruz, Julien Lancelot, Stéphanie Caby, Guilherme Oliveira, Raymond J. Pierce

**Affiliations:** ^1^Institut National de la Santé et de la Recherche Médicale U1019 – Centre National de la Recherche Scientifique UMR 8204, Center for Infection and Immunity of Lille, Institut Pasteur de Lille, Université de LilleLille, France; ^2^Genomics and Computational Biology Group, Fundação Oswaldo Cruz, Center for Excellence in Bioinformatics, Centro de Pesquisas René Rachou, National Institute of Science and Technology in Tropical DiseasesBelo Horizonte, Brazil

**Keywords:** epigenetics, schistosome, drug discovery, histone modifying enzymes, DNA methylation, microRNAs

## Abstract

The discovery of the epigenetic regulation of gene expression has revolutionized both our understanding of how genomes function and approaches to the therapy of numerous pathologies. Schistosomes are metazoan parasites and as such utilize most, if not all the epigenetic mechanisms in play in their vertebrate hosts: histone variants, histone tail modifications, non-coding RNA and, perhaps, DNA methylation. Moreover, we are acquiring an increasing understanding of the ways in which these mechanisms come into play during the complex schistosome developmental program. In turn, interest in the actors involved in epigenetic mechanisms, particularly the enzymes that carry out epigenetic modifications of histones or nucleic acid, as therapeutic targets has been stimulated by the finding that their inhibitors exert profound effects, not only on survival, but also on the reproductive function of *Schistosoma mansoni*. Here, we review our current knowledge, and what we can infer, about the role of epigenetic mechanisms in schistosome development, differentiation and survival. We will consider which epigenetic actors can be targeted for drug discovery and what strategies can be employed to develop potent, selective inhibitors as drugs to cure schistosomiasis.

## Introduction

Schistosomiasis is caused by flatworm parasites of the genus *Schistosoma*, five species of which infect humans in 74 tropical and sub-tropical countries. It is estimated that more than 230 million people are infected, of which 90% are in sub-Saharan Africa (Colley et al., 2014 for review). Nearly 30 years after its introduction the treatment and control of schistosomiasis relies almost exclusively on praziquantel, the only drug effective against all schistosome species infecting humans. Its use has been and remains an unquestionable success story; mass treatments of school-age children in sub-Saharan Africa under the Schistosomiasis Control Initiative (Fenwick et al., 2009) hold the promise of a marked continent-wide reduction in disease morbidity and mortality. Nevertheless, the massive use of this drug may well-lead to the selection of resistant/tolerant parasite strains. Episodes of drug tolerance have been reported (Doenhoff et al., 2008; Melman et al., 2009) and can be induced readily in the laboratory (Fallon and Doenhoff, 1994). In addition, changes to the local genetic polymorphism of parasites following treatment of the population have been detected (Norton et al., 2010; Coeli et al., 2013), suggesting an effect of drug selection pressure. Although the precise mechanism of action of praziquantel in killing schistosomes is unknown, its initial effects include the rapid influx of Ca^2+^ ions and calcium-dependent muscle contraction and paralysis (Day et al., 1992) and this may be mediated via its interaction with a calcium channel beta subunit (Kohn et al., 2001). However, resistance to praziquantel may be mediated by increased expression of the P-glycoprotein efflux pump, which is often involved in drug resistance mechanisms, following exposure to praziquantel (Messerli et al., 2009). Whether or not such reports are the harbingers of the development of resistance by schistosomes toward praziquantel, reliance on a single drug is patently untenable in the medium to long term.

Most of the current efforts to identify new drug leads for schistosomiasis and other neglected parasitic diseases rely on the screening of random compound libraries directly on the parasite maintained in culture (phenotypic screening). The recent publication of the genome sequences of a variety of parasites including the three main species of schistosomes that infect humans (Berriman et al., 2009; Zhou et al., 2009; Young et al., 2012) now means that approaches targeting specific gene products or pathways can be envisaged. These can include enzymes with activities specific to the parasite, or at least not found in the human host (e.g., Sayed et al., 2008), metabolic bottlenecks, or molecules that are targeted in other pathologies. For these a wide knowledge base and extensive libraries of inhibitors may already exist that can be exploited as starting points for the development of parasite-selective compounds. This type of approach also has the advantage that the molecular mechanism of action of a given compound, which is required for any new drug, is much easier to determine than with the random screening approach. However, both strategies are still used and both have proved fruitful sources of new drugs (Swinney and Anthony, 2011) although a more recent analysis of discovery of first-in-class drugs suggests a growing predominance of target-based approaches (Eder et al., 2014). Drug discovery is not a zero-sum game, but more a Nash equilibrium (Nash, 1950; Holt and Roth, 2004) in which the coexistence of strategies is not only possible but can also be highly productive.

Schistosomes are digenean parasites that successively infect freshwater snails (the intermediate host) and the vertebrate definitive host. They reproduce both asexually (within the snail host) and sexually (vertebrate host) and their life-cycle includes four distinct morphological forms and separate sexes at the adult worm stage (Colley et al., 2014). The complexity of schistosome development and differentiation implies a tight control of gene transcription at all stages of the life-cycle and that epigenetic mechanisms are likely to play a crucial role in these processes, suggesting that they are viable drug targets. In other pathologies, but most intensively in cancer, the targeting of epigenetic processes is increasingly exploited. Indeed, two histone deacetylase (HDAC) inhibitors have already been approved for use and a number of other candidate drugs are undergoing clinical trials (Arrowsmith et al., 2012). Moreover, large libraries of compounds that affect epigenetic actors are available for testing against parasites. Here, we will consider which epigenetic mechanisms can be targeted in schistosomes and what methodologies can be used to develop parasite-selective drug leads.

## Epigenetic mechanisms as drug targets

**Figure 1:**
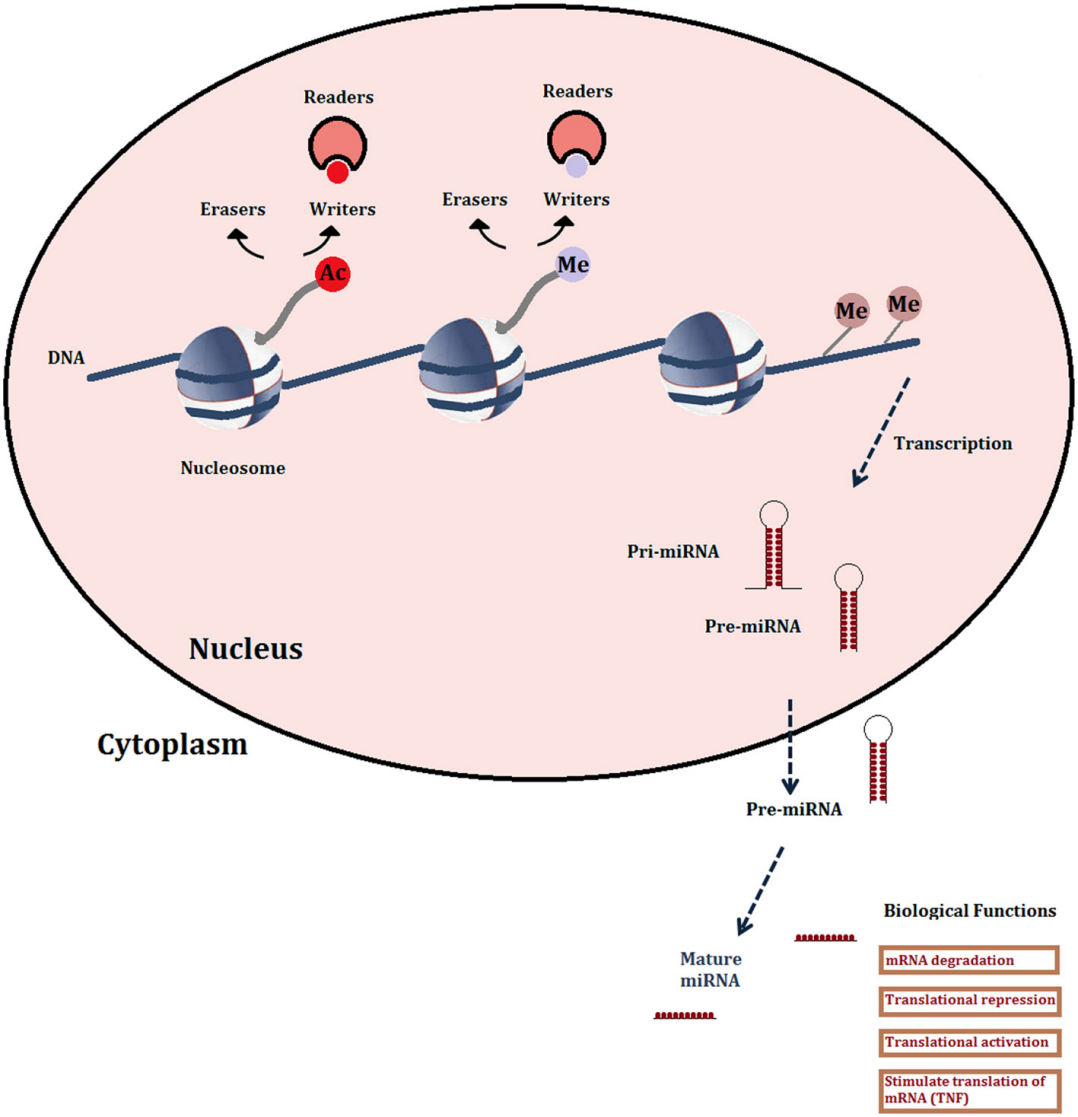
**Schematic representation of the major druggable epigenetic mechanisms**. Histone modifications shown are limited to acetylation and methylation since the enzymes (writers and erasers) and recognition domains (readers: bromodomains) are the most studied for drug development. Also shown are DNA methylation and microRNAs.

The term “epigenetics” envelops a variety of heritable changes in gene expression that are linked to structural modifications of the chromatin, without changes to the DNA sequence. These include DNA methylation, reversible post-translational modifications of histones, histone variants, chromatin remodeling factors and non-coding RNAs. Viewed as potential targets, the most readily “druggable” are the enzymes that carry out DNA methylation and histone modifications, and increasingly, micro-RNAs (miRNAs) among the non-coding RNA categories (Figure 1).

The investigation of the role of epigenetic mechanisms in the control of gene transcription in schistosomes, and hence in biological processes like development and reproduction, is in its early stages. Nevertheless, the knowledge so far acquired, or inferred from the nature of schistosomes as invertebrate metazoan organisms and from a detailed analysis of the epigenetic actors encoded in their genomes, can be exploited to develop novel therapeutic strategies. Moreover, insights into schistosome epigenetic mechanisms has been gained from studies aimed at developing such strategies, including for example the characterization of the actions of inhibitors of histone modifying enzymes (HMEs), or from transcript knockdown studies. Here we will review the current state of knowledge of the epigenetic apparatus in schistosomes, including the still disputed significance of DNA methylation, the miRNA repertoire, the histone modifying enzyme complement and the potential for the development of novel drug treatments targeting these elements.

## DNA methylation

DNA methylation encompasses the methylation or hydroxymethylation of cytosine residues, mainly, but not exclusively within CpG dinucleotides (Baubec and Schübeler, 2014) and is an important epigenetic mark associated with gene repression. In vertebrates three DNA methyltransferases (Dnmts) establish (Dnmt3a and Dnmt3b) and maintain (Dnmt1) DNA methylation marks. A further member of this family, Dnmt2 is primarily a tRNA methyltransferase with only weak DNA methyltransferase activity (Schaefer and Lyko, 2010). Disruption of DNA methylation patterns is present in a variety of diseases, particularly in cancer in which many oncogenic pathways lead to Dnmt1 overexpression, an overall DNA hypomethylation concomitant with hypermethylation of tumor suppressor genes at CpG islands in the promoter regions. Agents that provoke DNA demethylation, such as 5-azacytidine and 5-aza-2′-deoxycytidine (decitabine) have been approved for use in myelodysplastic syndrome (Yoo and Jones, 2006), but their mode of action is complex. It involves conversion to a triphosphate metabolite, incorporation into DNA provoking a DNA damage response and covalent trapping of Dnmt isoforms, followed by proteolysis of the Dnmts, demethylation and the reactivation of the hypermethylated genes (Streseman and Lyko, 2008). The cytotoxicity of the 5-aza nucleosides and the lack of a direct inhibitory effect on Dnmts have led to the search for leads for new drug development. One example is laccaic acid A, a recently developed direct DNA-competitive inhibitor of Dnmt1 (Fagan et al., 2013).

### DNA methylation in schistosomes

The presence of functional DNA methylation marks in schistosome genomes is controversial. Early work in which Southern blot analysis was carried out for selected genes after digestion with methylcytosine tolerant or sensitive restriction enzyme isoschizomers (*Hpa*II and *Msp*I) showed no differences in the restriction profiles for adult male or female *S. mansoni* DNA (Fantappié et al., 2001). Moreover, the methylcytosine-dependent restriction endonuclease McrBC failed to digest *S. mansoni* DNA. However, a more recent study (Geyer et al., 2011) in which a variety of more sensitive methods including GC-MS, anti-methylcytosine antibodies and targeted bisulfite sequencing were used concluded that cytosine methylation was indeed present and a hypermethylated repetitive intron within a forkhead gene was characterized. The only DNA methyltransferase encoded in the schistosome genome is Dnmt2. The methyltransferases usually associated with DNA methylation; Dnmt1 and Dnmt3 orthologs are both absent. Dnmt 2 has only weak DNA methyltransferase activity but has robust methyltransferase activity toward tRNA^Asp^ and other tRNAs (Goll et al., 2006). The diverse group of animal species (including *S. mansoni* and *Drosophila melanogaster*) that express only Dnmt2 have very low DNA methylation levels (Kraus and Reuter, 2011). However, Dnmt2 does retain some cytosine methyltransferase activity (Hermann et al., 2003) and Geyer et al. (2011) showed that siRNA knockdown of SmDnmt2 transcripts reduced overall methylcytosine levels in the schistosome genome. These authors have further suggested that cytosine methylation is conserved throughout the phylum Platyhelminthes (Geyer et al., 2013). Against this, a comprehensive study (Raddatz et al., 2013) using whole-genome bisulfite sequencing showed that the *S. mansoni* genome lacked a detectable DNA methylation pattern, even at the “hypermethylated” locus identified by Geyer et al. (2011). Some clusters of incompletely converted cytosines were detected outside this region, but were consistent with bisulfite deamination artifacts (Warnecke et al., 2002). However, although these results strongly suggested that the *S. mansoni* genome is in fact unmethylated, the criticism has been leveled that the life-cycle stage analyzed, adult male worms, has the lowest level of DNA methylation measured using an ELISA method (Geyer et al., 2013). Notwithstanding this controversy, which will only be resolved by genome-wide bisulfite sequencing of other life-cycle stages, Dnmt inhibitors were found to strongly affect adult worms, particularly in terms of the morphology of the ovaries and *in vitro* egg-laying (Geyer et al., 2011). Whether or not this is due to the inhibition of DNA or tRNA methylation, it does suggest that Dnmt inhibitors such as 5-azacytidine may provide the basis for developing precursors of novel anti-schistosome drugs.

## Micro-RNAs

Non-coding (nc) RNAs include many different classes of transcripts that do not code for proteins, but have various regulatory roles in transcription, stability or translation of protein-coding genes. Of these, miRNAs are the best characterized in terms of their functional roles and pathological implications, as well as therapeutic strategies targeting them (Ling et al., 2013). They are generated from long, capped and polyadenylated transcripts that are processed by a nuclear complex containing RNase III (Drosha: canonical pathway) or by the mRNA splicing machinery (non-canonical pathway) (Li and Rana, 2014 for review) into 60–100 nucleotide precursors that are then transported into the cytoplasm where they are processed by the RNase Dicer into mature, double stranded miRNAs (Figure 1). Classically, miRNAs regulate transcript levels through binding to the 3′UTR regions of their target mRNAs, usually resulting in translational inhibition or mRNA destruction. However, it is now clear that miRNAs may have other mechanisms of action, for instance increasing translation via the recruitment of protein complexes to the mRNA or by binding proteins that block translation (Elring et al., 2010). Different miRNAs have been shown to have either tumor-suppressive (e.g., miR-15a-mIR-16-1 cluster) or oncogenic (mIR-21, mIR-17-mIR-92 cluster, mIR-155) properties. Indeed, miRNAs can drive cancer: mIR 155 overexpression on its own provokes lymphoblastic leukemia or lymphoma in transgenic mice (Costinean et al., 2006). In cancer therapy, the upregulation of tumor-suppressive miRNAs has the advantage of simultaneously affecting a number of coding or non-coding genes that are targeted by the miRNA and that may be involved in the same or interacting pathways. A disadvantage is that a given miRNA may have different or even opposite effects in different cell types, depending on the expression patterns of its target genes. However, such considerations would be less of an obstacle in the therapy of parasitic diseases where it can be assumed that any disruption, positive or negative, of miRNA effects would be potentially deleterious to the parasite. Most current therapeutic strategies targeting miRNA in cancer are aimed at downregulating or blocking the function of oncogenic miRNA. One example consists in the use of antisense oligonucleotides, particularly those containing locked nucleic acids (LNA anti-mIRs) which are bicyclic RNA analogs in a locked configuration. One such compound, an anti-viral, miravirsen, is in clinical trials for the treatment of hepatitis C viral infection (Janssen et al., 2013; Lieberman and Sarnow, 2013).

### Schistosome miRNAs

A survey of the available *S. mansoni* EST sequences (Oliveira et al., 2011) concluded that 10.3% (21,107 sequences) match the genome but have no protein coding potential and are therefore possible ncRNAs. This in turn suggests that the parasite may use a range of ncRNAs in transcriptional and translational regulation. Moreover, the presence of proteins involved in miRNA processing (Drosha, Dicer, and Argonaute) (reviewed in Oliveira et al., 2011) supports a role for miRNA regulation of development and differentiation of schistosomes and explains the effectiveness of RNAi and siRNA knockdown of transcription in the parasite (Boyle et al., 2003). miRNAs were first identified in *S. japonicum* (Xue et al., 2008; Huang et al., 2009) in two separate studies that demonstrated the existence of a limited number of miRNA that are conserved in other organisms including humans and several hundred novel miRNAs. In the second study all the novel miRNAs (172) were identified by an inferred RNA hairpin and many were differentially expressed during the life-cycle (Huang et al., 2009). Deep sequencing studies (Hao et al., 2010; Wang et al., 2010) further showed the presence of large numbers of siRNAs derived from transposable elements, but also identified 38 novel *S. japonicum* miRNAs. In *S. mansoni*, the sequencing of a small-RNA cDNA library yielded 211 novel miRNA candidates of which 11 were further verified by Northern blotting (Simoes et al., 2011). Therefore, although further work is necessary to validate the schistosome specific miRNAs and determine which of them are shared between schistosome species, it is clear that these miRNAs are potential therapeutic targets. It is of note that flatworms show a gradual loss of *conserved* miRNAs during evolution (Fromm et al., 2013), which is suggested to be due to morphological simplification. However, they have equally gained specific ncRNAs, including endogenous siRNAs, which are differentially expressed during development, notably during the sexual differentiation of female worms (Cai et al., 2011; Sun et al., 2014). A therapeutic strategy based on LNA anti-mIRs would have the advantage of targeting parasite-specific sequences and hence avoiding off-target effects, but it is not yet known whether individual miRNAs could be valid therapeutic targets. There are several additional challenges associated with such a strategy (Ling et al., 2013), the main ones being to ensure bioavailability to the parasite and oral delivery, which would require a significant effort to investigate appropriate chemical substitutions.

## Post-translational modifications of histones

Histone post-translational modifications are currently under the most intensive study for drug development. The “writers” that add groups to histone N-terminal tails, “erasers” that remove them, or “readers” that recognize and bind them, are all potential therapeutic targets (Figure 1). The increasing variety of possible modifications includes phosphorylation, ubiquitinylation and sumoylation, but acetylation and methylation are the most abundant, most studied and their activity is mediated by the largest number of druggable proteins (Arrowsmith et al., 2012). Gene regulation is effected by combinations of these histone marks, leading to the “histone code” hypothesis (Strahl and Allis, 2000) whereby different chromatin states are defined by specific repertoires of marks.

Histone acetylation is a dynamic process regulated by histone acetyltransferases (HAT) that use acetyl-CoA as a co-factor and transfer an acetyl residue to the ε –amino group of lysines, particularly in the N-terminal tails of histones H3 and H4. The HDAC that remove this mark belong to four classes in mammals. Classes I, II, and IV have structurally-related catalytic domains and a Zn^2+^-dependent catalytic mechanism (Gregoretti et al., 2004). The class III HDACs, or sirtuins, are phylogenetically unrelated and rely on NAD^+^ as a co-factor (Greiss and Gartner, 2009). Histone acetylation neutralizes the positive charge of the lysine, leading to a more relaxed structure permitting recruitment of the transcriptional machinery and in consequence is associated with transcriptional activation.

Histone methyl marks are written on lysine or arginine residues in histone tails by S-adenosylmethionine-dependent methyltransferases and erased by two classes of demethylase, the Jumonji family of demethylases that are 2-oxoglutarate-dependent, or the flavin-dependent lysine-specific demethylase 1 (KDM1/LSD1) and 2 (KDM2/LSD2). Unlike acetylation, a methyl group has no effect on the overall charge of the lysine or arginine residue that carries it and the effects of the mark are mediated by “reader” proteins that either compact the nucleosomes or form complexes with other regulatory proteins. Moreover, lysine residues can react with different reader domains depending on their position and degree (mono-, di, -or tri-) of methylation and can consequently integrate signal platforms determining activation or repression of transcription (Badeaux and Shi, 2013).

The current list of inhibitors of “Histone Modifying Enzymes” (HMEs) approved for use in humans or in clinical trials reflects the initial concentration in this field on the HDACs as therapeutic targets. Of 18 such compounds, 17 are HDAC inhibitors (including the two approved compounds, Vorinostat, and Romidepsin) and another is a sirtuin (Sirt) 1 inhibitor (Arrowsmith et al., 2012; West and Johnstone, 2014). Of the HDAC inhibitors in clinical trials some inhibit class I and II enzymes indiscriminately, whilst others are more selective. Romidepsin preferentially inhibits the class I HDACs 1, 2, 3, and 8, whilst Vorinostat inhibits the class II HDAC6 and HDAC8 only poorly (Arrowsmith et al., 2012). Selectivity for a given HDAC or class may be of therapeutic importance as these enzymes have different targets. HDACs generally deacetylate both histones and other proteins. HDAC6 is not involved in epigenetic signaling at all but deacetylates tubulin and Hsp90 (Hubbert et al., 2002; Kovacs et al., 2005), while the only known HDAC8 substrate is SMC3, a component of the cohesin complex (Deardorff et al., 2012). HDAC inhibitors occupy the hydrophobic tunnel in the enzymes that accommodates the acetyllysine substrate and coordinate the zinc ion at the base of the tunnel, for example with a hydroxamate grouping as for Vorinostat. Selectivity can be based on differences between the make-up and architecture of the tunnel, or on the surface-accessible rim. However, within the cell HDACs are often part of multi-protein complexes that may alter substrate and inhibitor specificities compared to isolated recombinant proteins (Bantscheff et al., 2011) and this remark is likely even more pertinent in the case of other HMEs.

Of the seven mammalian NAD^+^-dependent class III deacetylases, or sirtuins (Sirt) Sirts 1, 2, 3, 6, and 7 have been shown to possess deacetylase activity (Feldman et al., 2012), although Sirt6 is also a fatty acylase (Jiang et al., 2013). The other two sirtuins, 4 and 5 are both predominately mitochondrial. Sirt5 is a demalonylase and desuccinylase (Du et al., 2011) and has recently been shown to regulate a novel lysine modification, glutarylation (Tan et al., 2014). Sirt4 exhibits ADP-ribosyltransferase activity (Haigis et al., 2006). Sirtuin inhibitors have been developed against Sirts 1, 2, and 3 that couple the deacetylation reaction with the cleavage of NAD^+^, liberating free nicotinamide. Inhibitors can bind either to the conserved NAD^+^-binding C-pocket, like nicotinamide itself, or the acetyllysine peptide-binding cleft between the large and small domains of the enzyme (Yuan and Mamorstein, 2012), or both. In addition, since Sirt1 expression has been associated with increased lifespan and memory, allosteric activators such as resveratrol have been explored as therapeutic agents Hubbard and Sinclair, 2014). Only one sirtuin inhibitor, selisistat, is currently in clinical trials, however, for Huntington's disease (Arrowsmith et al., 2012).

HAT generally lack obvious druggable sites and few selective inhibitors are currently available. The HAT catalytic domains have a conserved organization around a central fold where the acetyl-CoA cofactor binds. The peptide substrate binding site in the only solved structure is shallow and solvent accessible, reducing its capacity to be targeted by drugs (Arrowsmith et al., 2012). Among the inhibitors so far described are natural substances that promiscuously bind a variety of targets (Piaz et al., 2011), or isothiazolone covalent modifiers (Ghizzoni et al., 2009). These latter include the more recently developed pyridoisothiazolones that effectively inhibit cancer cell proliferation (Furdas et al., 2011). However, a potent, selective inhibitor of the HAT EP300, C646, has been developed that binds at the cofactor pocket and has pro-apoptotic effects on prostate cancer cells (Bowers et al., 2010). This indicates that at least certain HATs are valid, standalone therapeutic targets, but effective screening may depend on the reconstitution of multi-protein complexes in which they are active in the cell and which may modulate their enzymatic activity (Arrowsmith et al., 2012).

The protein methyltransferases include both lysine (KMT) and arginine (PRMT) methyltransferases that are phylogenetically unrelated but share the requirement for S-adenosylmethionine as a cofactor and a cofactor binding site adjacent to the channel that binds the peptide substrate (Arrowsmith et al., 2012). Both these sites can be used to generate selective and potent inhibitors for both PRMTs and KMTs (Spannhoff et al., 2009; Dowden et al., 2010). However, in order to screen certain of the latter enzymes, the reconstitution of protein complexes is a prerequisite. The KMT component of the PRC2 transcription repression complex, EZH2, which methylates H3K27, is inactive on its own and minimally requires the presence of at least two members of the complex, EED and SUZ12 (Helin and Dhanak, 2013). High-throughput screening has been carried out on a complex additionally containing AEBP2 and RbAp48 proteins and has yielded highly selective inhibitors such as GSK126, which is a promising lead for the treatment of lymphoma (McCabe et al., 2012). Complex recomposition is not always necessary; the H3K79 methyltransferase DOT1L requires no partner proteins and a highly selective inhibitor, EPZ-5676, has been developed using a structure-guided strategy, with significant activity in a rat xenograft model of MLL-rearranged leukemia (Daigle et al., 2013).

Histone demethylases are under increasing scrutiny as drug targets (Hojfeldt et al., 2013). Two unrelated families of proteins exert demethylase activity, the LSD family and the JMJC domain-containing demethylases. In mammals the LSD (for Lysine-Specific Demethylase) family comprises only two members, but they share an amine oxidase-like domain with several metabolic enzymes in addition to a SWIRM (SW13, RSC8, and Moira) domain that is only found in some chromatin-associated enzymes. These enzymes use FAD as a cofactor that binds to one of two folded subdomains of the amine oxidase-like domain, the other binding the substrate. However, substrate specificity may be regulated through interactions of the SWIRM domain with protein partners (Metzger et al., 2005). The conservation of the amine oxidase-like domain means that some inhibitors of monoamine oxidases, such as tranylcypromine also inhibit LSD1 (Schenk et al., 2012), however, selectivity for the latter can be improved in derivatives. One such inhibitor, ORY-1001, has greatly improved selectivity for LSD1 and is entering clinical trials (Maes et al., 2013). Few selective or potent inhibitors of JMJC (Jumonji C) demethylases have yet been reported. In these enzymes (31 family members in humans) the JMJC domain is catalytic and the enzymatic mechanism involves two cofactors, Fe(II) and 2-oxoglutarate, which are bound to it. Members of this family can demethylate mono-, di- or tri-methylated lysines. Most reported inhibitors contain metal chelating groups (analogous to HDAC inhibitors, but the latter are poor JMJC inhibitors) that compete with the 2-oxoglutarate cofactor. One remarkably selective inhibitor of JMJD3, an H3K27me3-specific demethylase, has been developed using a structure-guided approach (Kruidenier et al., 2012). This inhibitor, GSK-J1, interacts with critical amino acids involved in the binding of both the 2-oxoglutarate cofactor and the histone peptide substrate, and is competitive with the former but non-competitive with the latter. It is selective for both JMJD3 and Utx (a closely related JMJC demethylase active on the same substrate as JMJD3) but inactive against other members of the JMJC family. Moreover, GSK-J1 inhibits pro-inflammatory functions of human primary macrophages, indicating a possible therapeutic role. Moreover, a related JMJD3 inhibitor, GSK-J4, has recently been shown to have anti-tumorigenic activity against T-cell acute lymphoblastic leukemia (Ntziachristos et al., 2014).

Of the “readers” of epigenetic marks, bromodomain proteins, which read acetylated lysine residues, have so far attracted the most attention as drug targets. Bromodomains are composed of a characteristic antiparallel bundle of four α-helices that binds acetyllysine in a pocket at one extremity. They were first identified in the *Drosophila* gene brahma, hence the name, and in humans 61 bromodomains have been identified in 46 proteins, some containing more than one, and belong to eight distinct families (Hewings et al., 2012). Although the biological roles of most bromodomain proteins remain unknown, the connection of some of them with diseases such as cancer is becoming clearer (Arrowsmith et al., 2012; Filippakopoulos and Knapp, 2014). Small molecule inhibitors have been principally been developed against the BET bromodomain family. A triazolobenzidine (Nicodeme et al., 2010) was initially isolated as an inducer of ApoA1 expression and a derivative with enhanced activity in a reporter gene assay was only subsequently shown to interact with the bromodomains of BET family members BRD2, 3, and 4 using a chemoproteomic approach (Chung et al., 2011). Following an observation that thieodiazepines could bind to BRD4 a structure-based approach led to the development of a novel thieno-triazolo-1′4-diazepine named JQ1 (Filippakopoulos et al., 2011). This molecule binds to the BRD4 acetyllysine site and has reduced activity against other BRDs, but little or none toward other bromodomains. JQ1 induced a differentiation phenotype and growth arrest in a cell line derived from human squamous carcinoma. Since these founder studies, further selective inhibitors of BET bromodomains have been identified and clinical trials are ongoing, notably for JQ1.

### Schistosome histone modifications

An *in silico* analysis of the schistosome genome predictions and EST libraries (Anderson et al., 2012) showed that 21 of the 29 histone genes predicted in the genome are expressed in *S. mansoni*, the remainder being either unexpressed or having divergent sequences. Importantly, the N-terminal tails of the nucleosomal histones H3 and H4 are highly conserved, suggesting the functional conservation of the histone marks found in mammalian histones. The study of histone marks and their role in schistosome development and differentiation is still in its infancy, but studies involving inhibitors of HDACs and HATs have demonstrated the importance of histone acetylation and the interest of these enzymes as potential therapeutic targets. HDAC inhibitors including Trichostatin A (TSA) blocked the *in vitro* transformation of *S. mansoni* miracidia into primary sporocysts in a dose-dependent manner (Azzi et al., 2009) and this correlated with an increase in histone H4 acetylation. More recently, the same authors showed that differences in the levels of histone H3K9 acetylation on the promoters of genes encoding polymorphic mucins correlated with their differential expression in parasite strains compatible or incompatible with a given strain of the intermediate host, the freshwater snail *Biomphalaria glabrata*. TSA treatment ablated these strain-specific differences in expression (Perrin et al., 2013).

Histone acetyltransferase inhibition also has developmental consequences in schistosomes, particularly in egg maturation. The schistosome ortholog of the HAT GCN5 has been shown to acetylate H3 and H2A, and in particular H3K14 (de Moraes Maciel et al., 2008) and the CBP/P300 ortholog SmCBP1 primarily acetylates H4 (Bertin et al., 2006; Fantappié et al., 2008). Knockdown of either or both of these HATs in adult schistosomes has been shown to markedly reduce the transcription levels of the major eggshell protein p14 and to affect egg development. Moreover, these effects are reproduced by treating adult worm pairs with an HAT inhibitor, PU139 (Carneiro et al., 2014). After both inhibitor treatment and RNAi to knock down transcripts of the HATs, the phenotypic effects on egg laying and development were correlated with decreased acetylation of H3 and H4, increased methylation at H3K27, a marker of transcriptional repression, on the p14 proximal promoter.

The effects of both HDAC and HAT inhibitors on schistosomes suggest that histone acetylation may be a legitimate therapeutic target and this was further supported by a preliminary study showing that HDAC inhibitors like TSA and valproic acid could induce time and dose-dependent death of schistosomes (adult worms or schistosomula larvae) in culture (Dubois et al., 2009). Parasite death was associated with the induction of apoptosis in schistosomula shown by both TUNEL staining and the activation of the effector caspases 3/7. Once more, the molecular basis of these effects was evidenced by a global increase in histone H3 and H4 acetylation and significantly increased H4 acetylation at the proximal promoters of HDAC target genes, correlated with an increase in their transcription. More recently, similar effects have been observed using inhibitors of the class III HDAC, the sirtuins (Lancelot et al., 2013). Inhibitors of Sirtuins 1 and 2 such as salermide also induce apoptosis and death of schistosomula in culture. Moreover, salermide induces marked morphological alterations to the female worm genital apparatus, the arrest of egg-laying and the separation of worm pairs.

### Schistosome histone modifying enzymes: which are the best targets?

Schistosome HDACs and HATs are clearly potential drug targets, as are probably other HMEs, but there are several challenges that could potentially impede drug development:

- These enzymes are evolutionarily conserved, particularly their catalytic domains, and in order to avoid potential side effects, inhibitors that are selective for the schistosome enzyme have to be developed.
- If selective inhibitors can be developed, the targeted HME has to be essential to the parasite so that its inhibition is lethal. Many HMEs have overlapping specificities (Table 1) and the inhibition of one may be compensated by another. However, in the case of enzymes methylating (EZH) or demethylating (Utx) H3K27, only one isoform is present in schistosomes (Table 1), against two in humans, suggesting that these enzymes may represent particularly sensitive targets.

**Table 1:** **Identity and characteristics of *Schistosoma mansoni* histone modifying enzymes**.

**HME type**	**Class**	**Closest human ortholog**	**Size (aa)**	**Substrate specificity**	**Gene Id^a^**
HDAC	I	HDAC1	517^*^		Smp_005210
	I	HDAC3	418^*^		Smp_093280
	I	HDAC8	440^*^		Smp_091990
	II	HDAC4	291		Smp_191310
	II	HDAC5	701		Smp_069380
	II	HDAC6	1132		Smp_138770
	III (Sirtuin)	Sirt1	568^*^	H1 – H3 – H4	Smp_138640
	III (Sirtuin)	Sirt2	337^*^	H4K16	Smp_084140
	III (Sirtuin)	Sirt5	305^*^		Smp_055090
	III (Sirtuin)	Sirt6	386^*^	H3K9 – H3K56	Smp_134630
	III (Sirtuin)	Sirt7	517^*^		Smp_024670
HAT	GNAT	GCN5 (KAT2A)	899^*^	H3K9 – H3K14 – H3K18 H2B	Smp_070190
	GNAT	HAT1 (KAT1)	435	H4K5 – H4K12	Smp_178700
	MYST	Tip60 (KAT5)	463	H2AK5 – H3K14 – H4K5 – H4K8 – H4K12 – H4K16	Smp_053140
	MYST	MYST1 (KAT8)	496	H4K16	Smp_194520
	MYST	MYST2 (KAT7)	400	H4K5 – H4K8 – H4K12 – H3	Smp_171700
	MYST	MYST3 (KAT6A)	971	H3K14	Smp_131320
	CBP/p300	CBP/SmCBP1 (KAT3A)	2093^*^	H2AK5 – H2BK15 – H3K14 – H3K18 – H4K5 – H4K8	Smp_105910
	CBP/p300	CBP/SmCBP2 (KAT3A)	1892	H2AK5 – H2BK15 – H3K14 – H3K18 – H4K5 – H4K8	Smp_127010
	TAFII250	TFIID subunit 1	2241	H3 – H4	Smp_166840
HMT	SET	EZH1	1026	H3K27	Smp_078900
	SET	MLL3 (KMT2C)	399	H3K4	Smp_070210
	SET	MLL3 (KMT2C)	1560	H3K4	Smp_138030
	SET	MLL1/4 (KMT2D)	3002	H3K4	Smp_144180
	SET	MLL5 (KMT2E)	751	H3K4	Smp_161010
	SET	C20orf11/MLL5/Ranbp9	1305		Smp_009980
	SET	NSD2/WHSC1	1746	H3K4 – H4K20	Smp_160700
	SET	NSD1/2 (KMT3B)	1343	H3K36 – H4K44	Smp_137060
	SET	SET8 (KMT5A)	409	H4K20	Smp_055310
	SET	SUV 39H2 (KMT1B)	586	H3K9	Smp_027300
	SET	SUV4-20H1 (KMT5C)	613	H4K20	Smp_062530
	SET	SETD2	1575	H3K36	Smp_133910
	SET	SETD1B	1720/1822	H3K4	Smp_140390
	SET	SETDB	918/1032		Smp_150850
	SET	SETMAR	250	H3K9	Smp_043580
	SET	SET/MYND4	782		Smp_000700
	SET	SET/MYND4	527		Smp_124950
	SET	SET/MYND5	423/429/433		Smp_121610
	DOT1	DOT1L (KMT4)		H3K79	Smp_165000
	PRMT	PRMT1	252/359/334	H4R3	Smp_029240
	PRMT	PRMT3	1564		Smp_127950
	PRMT	PRMT4/CARM1	737	H3R2 – H3R17 – H3R26	Smp_070340
	PRMT	PRMT5	630	H2A – H4	Smp_171150
	PRMT	PRMT7	755		Smp_025550
HDM	KDM1	LSD1A	1043		Smp_150560
	KDM1	LSD1A	916		Smp_160810
	KDM1	LSD1 (KDM1)	1073	H3K4 – H3K9	Smp_162940
	JmjC	JMJD1B (KDM3)	273	H3K9	Smp_161410
	JmjC	JMJD2C (KDM4C)	1136	H3K9 – H3K36	Smp_132170
	JmjC	JMJD4	809		Smp_147870
	JmjC	JMJD6	839		Smp_137240
	JmjC	JHDM1D (KDM7)	653	H3K36	Smp_127230
	JmjC	Jarid (KDM5)	2372	H3K4	Smp_156290
	JmjC	jarid (KDM5)	1639	H3K4	Smp_019170
	JmjC	UTX (KDM6A)	1137	H3K27	Smp_034000

* Validated by cDNA cloning.

a Gene ID according to the genome annotation.

In consequence, for these particular therapeutic targets, an approach involving both target validation, notably by transcript knockdown (RNAi), and structural studies to determine specificities in the structure of the catalytic pocket, is essential.

The *S. mansoni* genome encodes 55 HMEs involved in protein acetylation/deacetylation or methylation/demethylation (Table 1) (Pierce et al., 2012). Some of these, including the class I HDACs (Oger et al., 2008) and the sirtuins (Lancelot et al., 2013) have been cloned and characterized and preliminary choices of targets can be made based on their degree of sequence conservation. In addition, the human orthologs of several of these enzymes are known to function only as part of a multiprotein complex as is the case for the H3K27 methyltransferase EZH2 mentioned previously (McCabe et al., 2012). High-throughput screening of the human enzyme has been done, using a five protein complex, but the resources devoted to carry out such a strategy for the development of anti-cancer therapies are not available in the case of neglected tropical diseases. Therefore, although the schistosome ortholog of EZH2 is a unique target, its screening will pose difficulties and it is therefore preferable to target an enzyme that is active on its own (like the H3K79 methyltransferase DOT1L for example Daigle et al., 2013). In addition, certain HMEs are very large proteins (Table 1) and although the production of truncated proteins containing the catalytic domain can be envisaged, this may affect both enzyme activity and the conformation of the catalytic pocket, limiting the relevance of screening or structural data.

These considerations reduce the choice of viable HME targets in schistosomes, but a large number remain. Two filters can be used to further limit the choice: the use of HME class inhibitors to determine whether enzyme families contain potential targets and transcript knockdown using RNAi to validate individual HMEs as stand-alone therapeutic targets. RNAi is still the only available means to achieve targeted knockdown of gene function in schistosomes, but its efficacy is transcript-dependent and phenotypes are not always observed (Stefanic et al., 2010).

### Development of selective inhibitors as drugs: the challenges

**Figure 2:**
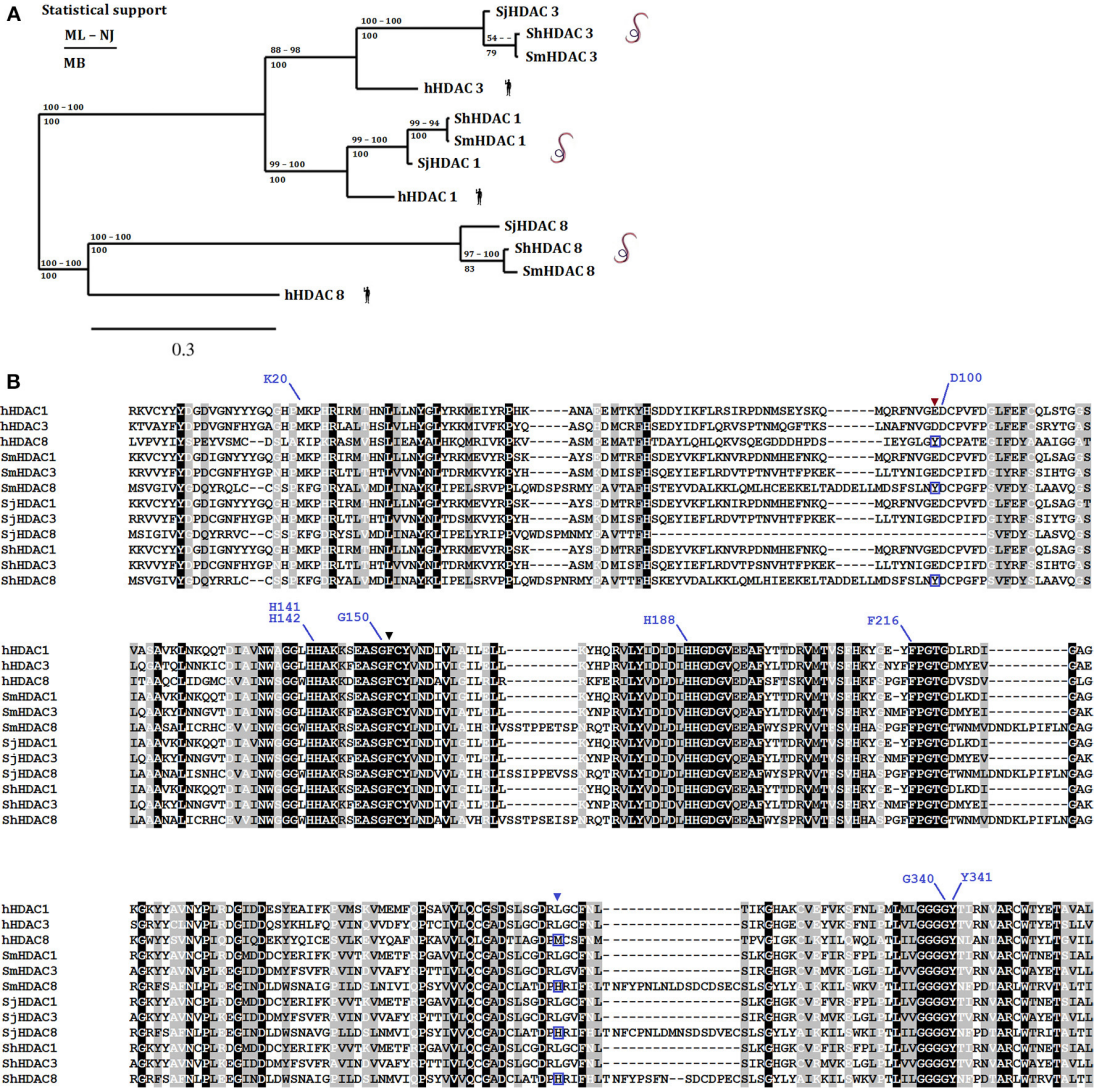
**Phylogenetic relationship amongst human and Schistosome class I HDACs**. Panel **(A)** displays a phylogenetic tree [ML, consensus from maximum likelihood; NJ, neighbor joining; and MB, Mr Bayes methods) built using the amino acid sequences from the catalytic domains of class I HDACs (HDAC1, 3, and 8) present in *S. mansoni* (Sm), *S. japonicum* (Sj), and *S. haematobium* (Sh) and their human (h) orthologs. Panel **(B)** shows an amino acid sequence alignment constructed with MAFFT with the class I HDACs used above. Conserved (black background and white letters), conservative changes (gray background and black letters) and less conservative changes (gray background and white letters) in amino acid positions are highlighted. Black (F151) and brown (Y99) triangles show residues that despite being conserved present conformational differences between human and Schistosomes HDAC8 (Marek et al., 2013). The blue triangle shows the position of the replacement of a methionine (M274) in the human HDAC8 by a histidine (H292) in SmHDAC8 (boxed amino acids below blue triangle). Residues that interact with the SmHDAC8 specific inhibitor J1075 are highlighted with blue lines.

An illustration of the strategy that can be employed to designate therapeutic targets is provided by the *S. mansoni* class I histone deacetylase 8 (SmHDAC8). Of the class I HDACs, SmHDAC8 was initially designated as a potential target for two reasons. First, transcript expression levels of SmHDAC8 are higher than those of SmHDAC1 and SmHDAC3 throughout the life cycle, notably in adult female worms (Oger et al., 2008). It is notable that HDAC 8 transcript levels are generally much lower than those of HDAC1 and 3 in normal human cells, but are markedly upregulated in some cancer cell lines and tissues (Nakagawa et al., 2007). Second, the analysis of the primary sequence of SmHDAC8 showed that it is less well-conserved compared to its human ortholog than the other two class I enzymes. This is demonstrated by the sequence alignment and phylogenetic analysis shown in Figure 2. The alignment shows that the essential residues for HDAC activity are conserved, but that the catalytic domain sequence contains insertions and substitutions that might indicate a change in architecture of the catalytic pocket, notably the replacement of a methionine (M274) in the human HDAC8 by a histidine (H292) in SmHDAC8. The status of SmHDAC8 as a stand-alone therapeutic target was enhanced by the use of generic HDAC8 inhibitors (unpublished results) and particularly by transcript knockdown using RNA interference (Marek et al., 2013). The latter showed that treatment of schistosomula with double-stranded RNA, followed by their injection i.v. into mice and harvesting of surviving worms 35 days later, led to a reduced worm recovery compared to mice treated with dsRNA encoding green fluorescent protein as a control.

Whilst molecular modeling seemed to show that only the charge difference within the catalytic pocket provided by His292 differentiated SmHDAC8 from its human counterpart, structural analysis by X-ray crystallography demonstrated a further important difference (Marek et al., 2013). In the schistosome enzyme, amino acid substitutions surrounding the catalytic pocket allow a change in the configuration of the side chain of phenylalanine 151. The side chain of the equivalent residue in human HDAC8, Phe152, adopts an obligatory flipped-in conformation that contributes to the narrow, hydrophobic tunnel accommodating the substrate or inhibitors. In contrast, in SmHDAC8 this side chain is free to adopt a flipped-out configuration, allowing the pocket to accommodate more bulky substrates or inhibitors. This difference, together with the charge difference, allows the possibility of identifying selective inhibitors for the schistosome enzyme. Indeed, an *in silico* screen, based on the crystal structure of SmHDAC8 and involving the docking of a large number of potential inhibitors, led to the identification of an inhibitor, J1075 (Marek et al., 2013), which had greatly improved selectivity for SmHDAC8 compared to human class I and II HDACs. Moreover, this inhibitor caused dose and time-dependent death of schistosome larvae in culture via the induction of apoptosis. Optimization of this and other inhibitors identified by this strategy is ongoing, and further potential drug precursors have been identified (Stolfa et al., 2014). It is also notable that the structural specificities of the SmHDAC8 enzyme compared to the human ortholog are shared with HDAC8 in other flatworms, including other schistosome species (Figure 2), *Echinococcus* sp. and *Clonorchis sinensis* (Marek et al., 2013). Therefore, drugs effective against *S. mansoni* may well be applicable to these other species.

This example proves the concept that individual epigenetic enzymes can be valid therapeutic targets, and that, even though these enzymes generally have conserved catalytic domains, sufficient differences in structure can exist to allow the development of selective inhibitors that are drug precursors. It remains to be seen, however, whether these inhibitors can be made sufficiently selective to preclude potentially harmful side effects and whether they can be developed into drugs useable in a single oral dose in humans.

## Conclusions

Epigenetic processes provide a wealth of potential therapeutic targets for the development of novel therapies against schistosomiasis and other parasitic diseases. The most readily exploitable of these targets are the HMEs, as well as perhaps Dnmt2, which lend themselves to target-based drug discovery strategies, necessary to ensure the development of parasite-selective drugs. A structure-based strategy has been initiated for *S. mansoni* HDAC8, involving the solution of the 3D structure of the catalytic domain and *in silico* docking of potential inhibitors. However, such enzymes can also be screened directly using random compound libraries and high-throughput methodologies using enzyme inhibition as the read-out. Moreover, the existing extensive libraries of HME inhibitors can be used for phenotypic screening of compound libraries for lethal effects on the parasite itself. In all these cases, since the molecular mechanism of action of the drug precursor is known, time will be saved in the process of optimizing selectivity.

The remaining potential targets discussed above, notably the histone modification readers, like bromodomain proteins, and miRNAs, require validation as effective targets and pose greater challenges for drug development. In order to identify the schistosome bromodomain proteins, an exhaustive analysis of the genomic data has still to be done. However, since both bromodomain proteins and miRNAs are under intensive investigation, particularly for the development of anti-cancer therapies, methodologies will be developed that could be exploited and adapted for the treatment of parasitic diseases.

Work on all the potential targets discussed here can benefit from the increasing knowledge base and compound libraries accrued, notably in the development of anti-cancer therapies. This “piggy-backing” approach (Dissous and Grevelding, 2010) holds great promise and can in part mitigate the relative lack of investment in efforts to improve the control and treatment of schistosomiasis and the other neglected parasitic diseases.

### Conflict of interest statement

The authors declare that the research was conducted in the absence of any commercial or financial relationships that could be construed as a potential conflict of interest.
